# Exosomal MicroRNAs in Pregnancy Provides Insight into a Possible Cure for Cancer

**DOI:** 10.3390/ijms21155384

**Published:** 2020-07-29

**Authors:** Preenan Pillay, Kogi Moodley, Manu Vatish, Jagidesa Moodley

**Affiliations:** 1 Pearson Institute of Higher Education, Faculty of Applied Science, Johannesburg 2153, South Africa; 2 Nuffield Department of Women’s and Reproductive Health, Women’s Centre, John Radcliffe Hospital, University of Oxford, Oxford 38655, UK; manu.vatish@wrh.ox.ac.uk; 3 Discipline of Human Physiology, School of Laboratory Medicine & Medical Sciences, College of Health Sciences, University of KwaZulu-Natal, Durban 4000, South Africa; MOODLEYKO@ukzn.ac.za; 4 Women’s Health and HIV Research Group, University of KwaZulu-Natal, Durban 4000, South Africa; jmog@ukzn.ac.za

**Keywords:** exosome diagnostics, cancer therapeutics, pregnancy, exosomal microRNA

## Abstract

The biological links between cancer and pregnancy are of recent interest due to parallel proliferative, immunosuppressive and invasive mechanisms between tumour and trophoblast development. Therefore, understanding “cancer-like” mechanisms in pregnancy could lead to the development of novel cancer therapeutics, however, little is understood on how tumour and trophoblast cells recapitulate similar molecular mechanisms. Based on our observations from a previous study, it was not only evident that exosomal miRNAs are involved in the pathophysiology of preeclampsia but also contained cancer-specific miRNAs, which suggested that “pseudo-malignant-like” exosomal-mediated mechanisms exist in pregnancy. The presented study therefore aimed to identify exosomal miRNAs (exomiR) in pregnancy which can be repurposed towards preventing tumour metastasis and immunosuppression. It was identified that exomiR-302d-3p, exomiR-223-3p and exomiR-451a, commonly associated with cancer metastasis, were found to be highly expressed in pregnancy. Furthermore, computational merging and meta-analytical pathway analysis (DIANA miRPath) of significantly expressed exomiRs between 38 ± 1.9 vs. 30 ± 1.11 weeks of gestation indicated controlled regulation of biological pathways associated with cancer metastasis and immunosuppression. Therefore, the observations made in this study provide the experimental framework for the repurposing of exosomal miRNA molecular mechanisms in pregnancy towards treating and preventing cancer.

## 1. Introduction 

Recent interest in the bioactive properties of exosomes in the pathophysiology of complex diseases such as cancer [1], pregnancy complications [2] and HIV [3] has been at the forefront of research and development. Exosomes are classified as nanovesicles ranging from a size of 50 to 120 nm and are released from the cell via the lysosomal pathway. They are characterised by a double-phospholipid membrane, encapsulating bioactive molecules comprising proteins and nucleic acids which have an important role in modulating key cellular physiological and pathophysiological processes [4]. More importantly, emerging scientific evidence indicates that exosomal-encapsulated miRNAs (exomiR) have a central role in targeting post-transcriptional modification processes in various pathologies [5], without, however, analysing the interrelated molecular mechanisms between cross-linked physiological and pathophysiological states. 

Pregnancy remains an immunological phenomenon whereby the mother can tolerate the semi-allogenic foetus for a defined time period through tightly regulated biological processes. Even though the exact mechanisms involved in this process have not been elucidated, the predominant mechanism is the ability of the placental syncytiotrophoblast cells to not only promote immune tolerance but also modulate migration and invasion into the maternal decidua [6]. Similarly, in cancer, tumour cells exhibit “trophoblast-like” cellular processes in achieving their proliferative, immunosuppressive and invasive properties [7,8,9,10,11,12]. Although the specific cellular molecular mechanisms in cancer and pregnancy are not completely understood, exosomes have been identified as a key constituent of the tumour and placental microenvironment (TME and PME). Their role in the TME and PME is to initiate and maintain multi-layered cellular communication directed towards cancer metastasis and trophoblast invasion during placental development, respectively [1,2]. 

The role of exosomes in modulating tumour metastasis has been extensively investigated [13] and found to exhibit consistent characteristics commonly associated with the pathophysiology of cancer. Tumour-derived exosomes (TEX) have been identified to have a key role in cellular signaling, tumour progression, metastasis and chemoresistance. Recent evidence indicates that this is achieved through the ability of TEX to re-programme their surroundings via their selectively packaged bioactive contents which (1) suppress immune responses for tumour evasion, and (2) promote metastasis and (3) chemoresistance. Despite the vast knowledge gained about TEX, their application as therapeutic targets remains limited due to the lack of understanding of their mechanistic and immunomodulatory role in cancer development. This could be overcome by understanding their function and mechanism of action in a natural non-malignant “tumour-like” model. The placenta has emerged as an ideal model to study intricate tumour mechanisms due to their overlapping biological processes [7,8,9,10,11,12] which could provide a novel perspective towards understanding the immunological maladaptation associated with cancer pathophysiology. 

More importantly the placenta has been shown to release exosomes (termed placental-derived exosomes) which are key factors involved in the immunomodulation of pregnancy and its related complications [14,15,16,17,18,19]. This has been mainly attributed to their physiological role in regulating foeto–maternal immune tolerance and the maternal metabolic adaptation to gestation [15,18]. In a recent publication, we reported that exosomes in maternal circulation contain microRNAs which are linked to biological pathways associated with cancer [19]. Our findings suggest that exosomes from trophoblast and tumour cells have the ability to create a microenvironment which supports immunologic privilege and angiogenesis through cellular reprogramming mechanisms which regulate hyper-proliferation, invasion, angiogenesis and immune-evasion [19]. 

Due to the similar roles of exosomes in pregnancy and cancer, the presented study therefore aims to compare and integrate the interconnected biological processes of exomiRs in pregnancy to cancer biology, with the objective of identifying possible interrelated biological pathways which can be used to develop cancer treatment. This would provide a novel perspective towards understanding the immunological maladaptation associated with cancer pathophysiology. 

## 2. Results and Discussion

### 2.1. Participant Clinical Characteristics 

Clinical characteristics of normotensive pregnant women (N) enrolled in this study are shown in Table 1 below. There were no significant differences in blood pressure, BMI and age between the N (≤33 weeks) and N (≥34 weeks) groups. 

### 2.2. Isolation and Characterisation of Exosomes in Maternal Circulation

**Figure 1:**
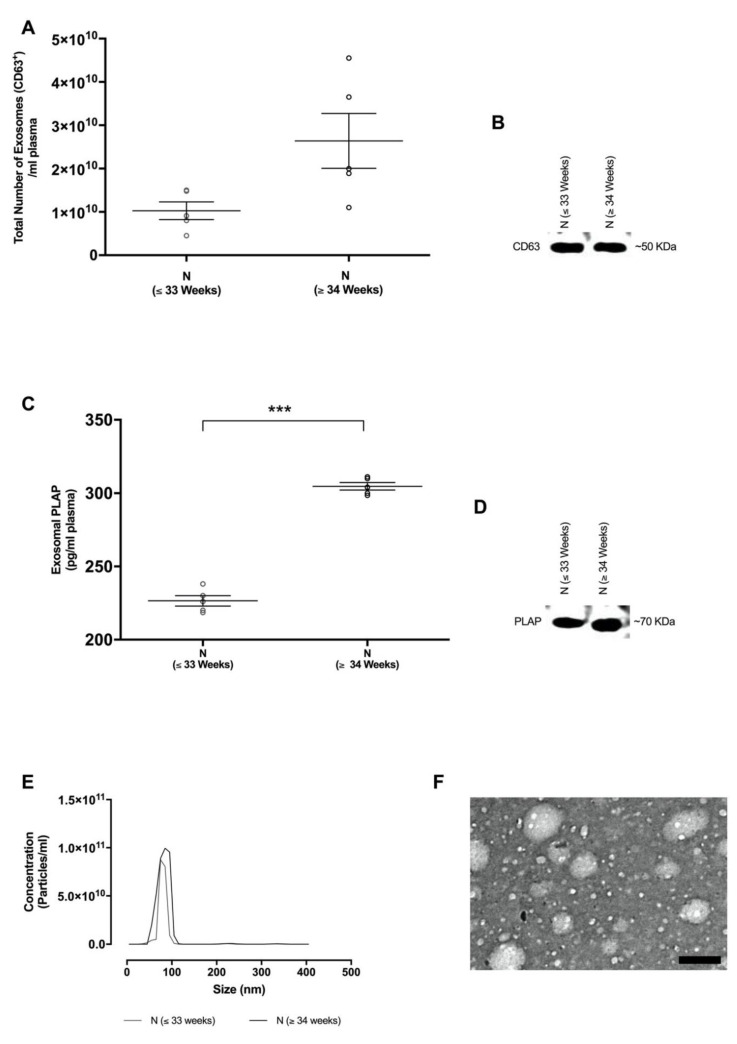
Identification and characterisation of exosomes from normotensive pregnant women. (**A**) Total exosome concentration was determined by the quantification of the exosomal CD63 marker per mL of plasma using ELISA. (**B**) Western blot analysis for the CD63 exosome enriched marker. (**C**) Placenta-derived exosomes (i.e., exosomal placental alkaline phosphatase (PLAP) was quantified per mL of plasma by ELISA. (**D**) Western blot analysis for the exosomal PLAP marker. (**E**) Nanoparticle tracking analysis (NTA) illustrating the representative vesicle size distribution (nm). (**F**) Electron micrograph of isolated exosomes, scale bar 100 nm. In **A**,**C**, the data are expressed as aligned dot plots and values are mean ± SEM. *** *p* < 0.0001 N (≤33 weeks) vs. N (≥34 weeks).

Isolated exosomes from each group were analysed for size distribution using nanoparticle tracking analysis & transmission electron microscopy and were found to be within the accepted exosomal size range (20–130 nm, Figure 1E,F). The total number of exosomes in maternal circulation was quantified using an exosome validated ELISA kit which detected the exosome specific CD63 marker. Total exosome concentration in N ≤ 33 weeks (1.02 ± 0.2 × 10^10^ total exosomes/mL, *p* < 0.0001) and N ≥ 34 weeks (2.36 ± 0.32 × 10^10^ total exosomes/mL, *p* < 0.0001) were obtained. Moreover, Western blot analysis was positive for the exosomal CD63 marker in all samples (Figure 1B). 

In order to determine the contribution of placental-derived exosomes present in maternal plasma, the placental alkaline phosphatase (PLAP) content per exosomal fraction was determined using ELISA (Figure 1C). A significant increase in placental-derived exosomes was observed in N (≥34 weeks) in comparison with N (≤33 weeks) (304.7 ± 5.7 vs. 226.5 ± 7.92 pg/mL, *p* < 0.0001). Furthermore, isolated exosomes were positive for the membrane-bound PLAP marker by Western blot analysis (Figure 1D).

### 2.3. Exosomal miRNA Expression in Pregnancy and Their Links to Cancer Metastasis and Immunosuppression 

Analysis of exomiR data obtained from normotensive pregnant woman (30 ± 1 weeks; *n* = 5 and 38 ± 1 weeks; *n* = 5) reveals the interconnected biological properties of exosomes in pregnancy in relation to cancer-like biological processes. A total of three exomiRs with a Log_2_ expression level greater than and equal to 5.5 were identified for further individual analysis (*p* < 0.001; Table 2). Each selected exomiR was then evaluated for their biological function using DIANA-mirPath (v.3.0), Kyoto Encyclopedia of Genes and Genomes (KEGG), microT-CDS (v5.0) target analysis in pregnancy (Table 2).

#### 2.3.1. Exosomal hsa-miR-451a

Exosomal hsa-miR-451a was found to be the highly expressed in pregnant woman (Log_2_; N 30 weeks: 11.06 ± 0.03 and N 38 weeks: 9.95 ± 0.01, Table 2) and has a function in the estrogen signaling pathway (KEGG: hsa04915, *p* < 0.005) by targeting ATF2, a key protein involved in transcription factor regulation. Although miR-451a regulation of ATF2 has not been validated in pregnancy, novel ATF2 isoforms were identified within the myometrium, with a role in inducing uterine contractions during labor [20,21,22,23]. Moreover, in cancer, ATF2 has been recently found to: (1) have tumour-suppressive properties as its reduced expression has been shown to promote tumourgenesis [24] and (2) enhance the efficacy of chemotherapeutics [25]. Therefore, the identification of exomiR-451a in pregnancy and cancer [26] suggests that parallel exosomal-mediated mechanisms involved in controlling ATF2 expression during placental and tumour development exist. Importantly, miR-451a has been validated to have direct tumour-suppressive functions by targeting: (1) MIF, c-MYC and AKT1 which attenuate AKT/mTOR pathway activation to inhibit cancer metastasis and (2) LPIN1 expression to inhibit tumourigenesis through the control of tumour cell apoptosis and angiogenesis [26]. This would suggest that placental tumour-like mechanisms could be manipulated to control tumour metastasis. 

#### 2.3.2. Exosomal hsa-miR-302d-3p

Exosomal hsa-miR-302d-3p was highly expressed in pregnant woman (Log_2_; N 30 weeks: 7.54 ± 0.21 and N 38 weeks: 9.24 ± 0.81, *p* < 0.05, Table 2) and targets the estrogen signaling (KEGG: hsa04915, *p* < 0.05) and proteoglycans in cancer pathways (KEGG: hsa05205, *p* < 0.05). Although these target pathways of miR-302d-3p have not been validated in cancer and pregnancy, the role of exomiR-302d-3p in the proteoglycans in the cancer pathway directly links exosomal molecular processes in pregnancy to cancer. Proteoglycans in cancer have a pivotal role in tumour metastasis by modulating tumour cell growth, survival, angiogenesis, migration and invasion [27]. This is due to their ability to regulate neovascularisation and neoplastic growth through cellular receptor and ligand interactions [27]. Similarly, in pregnancy, proteoglycans have a profound effect on the placental development, remodeling and growth of the endometrium by targeting angiogenic pathways [28]. Hence, the aberrant dysregulation of proteoglycan synthesis has been frequently associated with pregnancy-related complications [29,30] and tumour metastasis [27,31]. Therefore, the identification of exomiR-302d-3p in pregnancy suggests an exosomal mechanism of cancer-like proteoglycan regulation which could be repurposed in cancer to control proteoglycan synthesis within the TME. 

Importantly, proteoglycans in pregnancy have a role in suppressing alloreactivity at the foeto–maternal interface which ensures appropriate foeto–maternal immune tolerance [32]. Thus, the aberrant expression of proteoglycans has been commonly associated with inappropriate allogenic responses which cause foetal rejection. Similarly, in cancer, proteoglycans have an immunoregulatory role whereby TME-associated proteoglycans have the ability to promote anti-tumour immunity by blocking TGF-β1 signaling and limiting IL-10 release [27,33]. Additionally, TME proteoglycans have been shown to initiate metastatic signaling cascades in the promotion of tumour immune evasion [27]. Although the exact immunosuppressive mechanisms of proteoglycans are unknown, our findings suggest that exomiR-302d-3p in pregnancy may have a central role in regulating allogenic reactivity by controlling proteoglycan synthesis which in turn may increase T- cell recognition of allogenic MHC complexes at the foeto–maternal interface. Thus, simulating similar mechanisms in cancer could enhance our understanding on how to manipulate tumour immunosuppressive responses in preventing cancer metastasis. 

Although exomiR-302-3p has not been validated to directly target the estrogen signaling pathway in pregnancy and cancer, it has been shown that estrogen receptor 1 (ESR1), a target of miRNA-302-3p, has a role in both physiological and pathological states. Estrogen receptor 1 has a role in cancer metastasis whereby mutations of this gene have been associated with alterations in tumour adhesive and migrative properties [34,35,36]. In pregnancy, ESR1 is known to modulate the function of estrogen towards maintaining successful parturition [37,38], thus our findings suggest that exomiR-302-3p may regulate trophoblast differentiation by targeting estrogen signaling pathways. Therefore, understanding the exomiR-302d-3p-mediated regulation of ESR1 and other associated genes could provide a better understanding of the mechanisms involved in parturition which can be repurposed towards developing targeted cancer therapy. Moreover, miRNA-302b-3p targets transcription factor ATF6B which is involved in the unfolded protein response (UPR) during ER stress in the estrogen signaling pathway [39]. In pregnancy, ATF6B is known to modulate PlGF transcription in trophoblast cells [40], which is a central factor involved in maintaining placental angiogenesis and vasculogenesis. Similarly, in cancer, ATF6B has been identified to be involved in regulating the UPR, whereby chronic activation of this response is associated with tumour metastasis [41]. Therefore, since the UPR is a precursor to ER stress [42], the identification of exomiR-302-3p in pregnancy suggests its role in controlling placental ER stress responses. Since similar UPR mechanisms are involved in placental and tumour development, it may therefore be hypothesised that UPR-associated molecular interactions of exomiR-302-3p identified in pregnancy can be remodeled in cancer to prevent tumour metastasis.

#### 2.3.3. Exosomal hsa-miR-223-3p

Exosomal hsa-miR-223-3p was highly expressed in pregnant woman (Log_2_; N 30 weeks: 4.95 ± 0.1 and N 38 weeks: 5.5 ± 0.03, Table 2) and is involved in the mucin-type O-glycan biosynthesis pathway (KEGG: hsa00512, *p* < 0.05) by targeting GALNT7 and GALNT18. This pathway is responsible for the synthesis of mucin O-glycans which are highly O-glycosylated antigenic glycoproteins (Tn, sialyl-Tn and disialyl- Tn antigens) commonly found in mucous secretions on cellular surfaces and bodily fluids. There are no known validated targets of miRNA-223-3p linked to mucin-type O-glucan synthesis in cancer and pregnancy. However, in pregnancy, the expression of exomiR-223-3p suggests that exosomes may control allogenic reactivity by regulating T and Tn antigen glycosylation which in turn maintains the foeto–maternal immune balance. This is achieved by the ability of MHC-I and MHC-II to present the modified Tn antigens which have undergone post-transcriptional modifications to enable T-cell recognition at the foeto–maternal interface [43,44]. Whilst exomiR-223-3p has been found to be upregulated in metastatic cancer, the exact regulatory mechanisms involved remain unknown. However, the results presented in this study suggest that exosomal encapsulation of miRNA-223-3p may be a predominant mechanism in Tn, sialyl-Tn and disialyl antigen biosynthesis required for foeto–maternal immune tolerance. Similar mechanisms may exist in cancer, whereby exomiR-223-3p contributes to the TME in controlling immunity towards the prevention or initiation of metastatic growth. Thus, remodeling pregnancy-associated exosomal mechanisms of miR-223-3p encapsulation and activity could lead to the development of targeted cancer treatment.

Although there is no direct interaction of miRNA-223-3p with glycosylation pathways, it has been proven to target protein expression levels in pregnancy [45] and cancer [46]. In pregnancy, miRNA-223-3p regulates endometrial receptivity to blastocyst implantation by targeting leukemia inhibitory factor (LIF), which modulates biological processes such as decidualisation, embryo–endometrial interaction, blastocyst growth, trophoblast invasion and immune modulation [45]. In cancer, LIF has a central role in regulating CD8^+^ T-cell tumour infiltration which stimulates protumoural-associated macrophages [47] and thus remains an important target for preventing tumour metastasis. However, targeting LIF for effective onco-therapeutics is limited due to its unknown regulatory mechanisms. Whilst exomiR-223-3p has not been validated to target LIF in cancer, it has been shown to suppress tumour metastasis, hence these findings could provide further insight into understanding the role of exomiR-223-3p in LIF expression. Since exosomal encapsulation of miRNA-223-3p in pregnancy may provide a possible mechanism of targeting mucin-type O-glycosylation [19], similar mechanisms can be recapitulated in cancer for the control of cellular processes and immune responses to prevent tumour metastasis and immune evasion.

### 2.4. Differential Exosomal miRNA Expression in Pregnancy and Their Links to Cancer Metastasis and Immunosuppression

**Figure 2:**
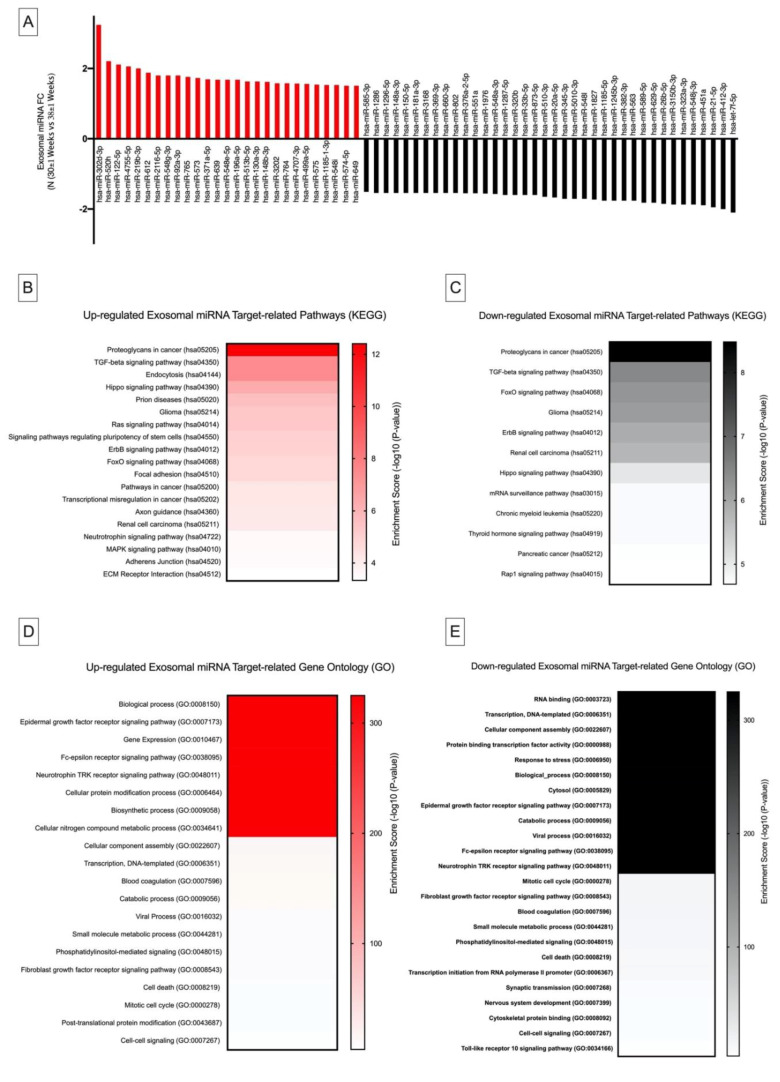
Differential DIANA miRPATH (V3.0) expression analysis of exosomal miRNA in N (30 ± 1 Weeks) vs. N (38 ± 1 Weeks). (**A**) FC values of differentially expressed miRNA in N (30 ± 1 Weeks) in comparison with N (38 ± 1 Weeks). Kyoto Encyclopedia of Genes and Genomes pathway mapping for top canonical pathways of differentially (**B**) **■ ** upregulated and (**C**) **■** downregulated exosomal miRNA. Gene Ontology enrichment analysis of (**D**) **■ ** upregulated and (**E**) **■** downregulated exosomal miRNA.

Differential expression of exomiRs between 38 ± 1.19 and 30 ± 1.11 weeks of gestation was analysed using the nSolver Software (V 4.0). Fold changes were transformed into log 2 scale (Log_2_ FC). Statistically significant exomiRs were selected based on an arbitrary |Log_2_ FC| ≥ 1.5 (Figure 2A, Supplementary Materials Table S1). To evaluate the possible function of the significantly altered exomiR expression levels between 38 ± 1.19 vs. 30 ± 1.11 weeks of gestation and their possible links to cancer biological processes, KEGG pathway and Gene Ontology (GO) target enrichment analysis was conducted. This was performed using DIANA mirPATH and the microT-CD (V5.0) database (Figure 2B–E). Differential expression of significant exomiRs between 38 ± 1.19 vs. 30 ± 1.11 weeks of gestation revealed exosomal-linked GO gene function and KEGG pathways which are closely linked to cancer metastasis and immunosuppression.

#### 2.4.1. Kyoto Encyclopedia of Genes and Genomes Pathway Analysis

Kyoto Encyclopedia of Genes and Genomes analysis indicated that the significantly upregulated and downregulated exomiRs (Figure 2B and C, respectively, *p* ≤ 0.001) between 38 ± 1 vs. 30 ± 1 weeks of gestation targeted the proteoglycans in the cancer pathway (hsa05205, *p* ≤ 0.05). Proteoglycans in cancer and pregnancy were previously discussed in Section 2.3.2. However, emerging evidence indicates that proteoglycans are involved in innate immunity by serving as endogenous ligands of toll-like receptors and activating intracellular inflammasomes which trigger an inflammatory response [48,49]. Therefore, the observed regulation of specific exomiRs in normal pregnancy (Figure 2A) suggests that exosomes may possibly be involved in controlling “cancer-like” proteoglycan pathways for appropriate cellular and immune-modulatory processes to occur during pregnancy. These findings indicate that similar mechanisms may have a modulatory role in cancer. Therefore, the identified set of exomiRs in pregnancy could be replicated in an engineered model to target cancer proteoglycan pathways towards preventing tumour metastasis. 

#### 2.4.2. Gene Ontology Analysis

Gene Ontology computational analysis of significantly upregulated and downregulated exomiRs (Figure 1D and E, respectively, *p* ≤ 0.001) between 38 ± 1 vs. 30 ± 1 weeks of gestation targets epidermal growth factor receptor signaling (GO:0007173, *p* ≤ 0.001). In pregnancy, epidermal growth factor receptor signaling (EGFR) plays a role in the regulation of decidualisation and trophoblast differentiation which is required for the implantation and maintenance of pregnancy [50,51]. Previous studies suggest that EGFR bound to ligands converge with other signaling pathways to form a signaling network for the control of growth factors, cytokines and inflammatory mediators which are responsible for maintaining trophoblast homeostasis. Similarly, in cancer, EGFR drives tumourigenesis through non-canonical mechanisms involved in stress-induced trafficking, autophagy and energy metabolism [52]. Thus, the controlled regulation of exomiRs in pregnancy reveals an exosomal-modulated EGFR homeostatic response which can be repurposed towards understanding non-canonical EGFR signaling mechanisms in cancer metastasis.

Interestingly, GO pathway analysis of both up- and downregulated exomiRs (Figure 1D and E, respectively, *p* ≤ 0.001) between 38 ± 1 vs. 30 ± 1 weeks of gestation indicates their role in epsilon receptor signaling (GO:0038095, *p* ≤ 0.001). Fc epsilon receptor is known to have a high affinity for IgE and is involved in the transport of IgE across the placenta and cytokine activity [53,54,55,56]. Even though the role and mechanism of IgE transport remains unclear, the ability of IgE to bind to Fc epsilon receptor on APCs enhances antigen presentation in pregnancy [57] which could present a route of priming the foetal immune system to maternal allergens. This process is enhanced in the second trimester of pregnancy [57], suggesting that the foetus develops its immune system during this time, consequently leading to the local production of cytokines which contributes to the development of the foetal immune system. Similar mechanisms may exist in cancer since recent novel concepts for cancer immune therapy suggest that IgE antibodies can be used to facilitate immune activation through the enhanced interaction of Fc epsilon receptors which is central in the stimulation of the immune responses directed towards overcoming tumour immune suppression and increased potency and longevity of immune surveillance against tumours [58]. Since the analysis of exomiRs in pregnancy were shown to target Fc epsilon receptor signaling, we propose that placental-derived exosomes facilitate antigen presentation towards enhancing foetal adaptive immunity to maintain foeto–maternal immune tolerance by controlling allergy inflammatory responses. This presents a novel type of exosomal-mediated immune surveillance during pregnancy which controls concomitant immune mechanisms required for foeto–maternal immune tolerance. This molecular mechanism coincides with cancer biology [58], thus simulating and manipulating similar high-affinity cognate Fc receptor exosomal signaling mechanisms in tumour cells that could serve as a viable target for tumour recognition and concomitant immunity towards natural, potent, long-lived cancer immune surveillance.

### 2.5. Future Experimental Framework for the Repurposing of Exosomal miRNA Identified in Pregnancy towards a Cure for Cancer

The TME consists of a network of stromal cells, endothelial cells, vasculature, fibroblasts, nerve cells and immune cells as well as the extracellular matrix which can either support non-malignancy through tumour-suppressive properties or mechanistically modify the TME to promote metastasis [59,60]. Similarly, in pregnancy, the placental microenvironment (PME) consists of a network of trophoblast cells, vasculature, immune cells and extracellular matrix which co-ordinate a plethora of biological and cellular functions towards ensuring foeto–maternal immune balance [61,62]. Since exosomes are known to be key constituents of the TME and PME, the findings from this study illustrate the probability that pregnancy-associated exomiRs contribute towards a cancer-like TME to ensure survival of the semi-allogenic fetus. It is therefore suggested that from the data analysed, exosomal mechanisms in pregnancy are the missing parallel links required to understand malignancy as an immunodynamic state and how the host’s immune system can be manipulated to recognise tumour-specific antigens as non-self, thereby leading to immune activation and prevention of metastasis. 

**Figure 3:**
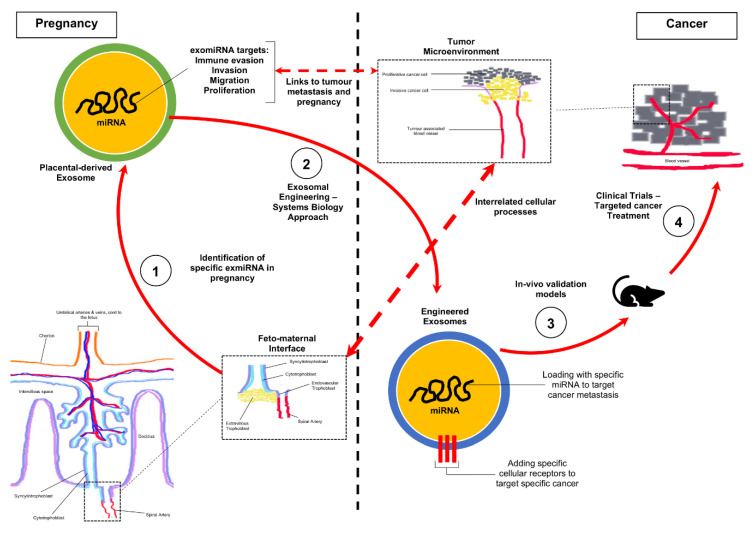
Strategy for the repurposing of pregnancy-associated exomiR for the treatment of cancer. Schematic representation of the development of targeted cancer therapeutics using interrelated exomiR biological mechanisms identified in pregnancy. (1) Our study has identified novel exomiR in pregnancy which is linked to cancer biological processes such as immune evasion, migration and proliferation. (2) We propose that these specific targets can be used to engineer exosomes which target cancer cells to correct biological processes in preventing metastasis. (3) Preclinical trials which will involve testing in multiple in vivo models to assess efficacy in delivery and tumour cell reprogramming. (4) Clinical trials to determine oncological efficacy and interaction other human biological processes.

Based on the analysis of the data presented in this study, our future work will be focused on developing novel treatment methods for cancer by repurposing pregnancy-associated exomiRs (Figure 3). In brief, this will be conducted in phases which would involve: (1) identification of exomiR in pregnancy and their specific targets in relation to cancer biological processes, (2) bioengineering exosomes to contain selected pregnancy-associated miRNA to (a) prevent tumour cells from evading the host’s immune system by co-opting certain immune responses and (b) preventing metastasis by targeting protein synthesis, through the modification of the TME, (3) validate functions using in vivo murine and primate models and (4) conduct clinical trials to validate efficacy. 

## 3. Materials and Methods

### 3.1. Ethics Statement

Ethical and institutional approval were obtained from the Biomedical Research Ethics Committee of the University of KwaZulu-Natal (BE310/15), South Africa. Participants were recruited from the Prince Mshiyeni Hospital, Kwa-Zulu Natal, South Africa, via written informed consent. The study was conducted in accordance with the Declaration of Helsinki.

### 3.2. Study Group

Normotensive pregnant woman (30.11 ± 1.56 weeks; *n* = 5 and 38 ± 1.9 weeks; *n* = 5) were identified with a blood pressure of 120 ± 10/80 ± 5 (systolic/diastolic mm Hg) with absent proteinuria (analysis via urine dipstick test, Medinox, Gauteng, South Africa). All recruited women had singleton pregnancies with no evidence of infections, surgical, medical or other obstetric complications. Blood from maternal circulation was collected and the plasma samples were stored at −80 °C for analyses (BD Vacutainer Tubes (EDTA), Becton Dickinson and Company, Gauteng, South Africa).

### 3.3. Exosome Isolation 

The exosome miRCURY kit (Qiagen, Hilden, Germany) was used to isolate exosomes as per the manufacturer’s instructions with minor modifications as previously described [14]. Briefly, plasma was centrifuged at 300 g for 10 min. Thereafter, the supernatant was centrifuged at 2000 g for 30 min to remove dead cells. The supernatant was recovered and centrifuged at 12,000 g for 45 min to remove cell debris. The supernatant was thereafter passed through a 0.22 µm spin column (Corning, NY, USA) to remove unwanted cells and debris. De-fibrination was achieved by adding thrombin to the filtrate followed by centrifugation at 10,000 g for 5 min. The supernatant was recovered and precipitation buffer was added and incubated for 4 h at 4 °C. After the incubation period, exosomes were recovered by centrifugation at 500 g for 5 min at 20 °C. The final pellet containing exosomes was resuspended in resuspension buffer for further analyses. Exosomal total protein concentration was determined using the RC-DC Protein Assay as per the manufacturer’s instructions (Bio-Rad Laboratories, Hercules, CA, USA). 

### 3.4. Exosomal RNA Isolation

Exosomal RNA was isolated with the total exosome RNA isolation kit (Life Technologies, CA, USA) as per the manufacturer’s instructions. Total RNA concentration was measured using the NanoDrop 2000 spectrophotometer (Thermo Scientific, MA, USA). 

### 3.5. Exosome Characterisation 

#### 3.5.1. Nanoparticle Tracking Analysis

Nanoparticle tracking analysis was used to quantify and determine the size distribution of the isolated exosomes as described in our previous publication (NanoSight500 NTA 3.0 Nanoparticle Tracking Analysis Release, Version Build 0069, Salisbury, UK) [14]. In Brief, samples were diluted in PBS to obtain a particle distribution of 10 and 100 particles per image. The following script was inputted into the instrument for analysis: PUMPLOAD, REPEATSTART, PRIME, DELAY 10, CAPTURE 60, REPEAT 5. The videos were recorded at a camera level of 10, a camera shutter speed of 20 ms and camera gain of 600, which were kept consistent between samples. The recorded videos were analysed to obtain the mean particle size and concentration of particles. Exosomal particle size is represented as the mean particle size ± SEM.

#### 3.5.2. Western Blotting

Exosomes were lysed with 1X RIPA buffer at room temperature. Thereafter, the protein content was determined using the RC-DC Protein Assay (Bio-Rad Laboratories, Hercules, CA, USA). Samples were thereafter incubated for 5 min at 95 °C with Laemmli buffer (2X). Thirty-five micrograms of exosomal protein was added to each lane of the sodium dodecyl sulfate-polyacrylamide gel electrophoresis (SDS-PAGE) and run at 150 volts. Thereafter, the protein on the SDS-PAGE gels were transferred onto polyvinylidene fluoride membranes (PVDF) and probed with the primary antibody, CD63 (ExoAB, System Biosciences, CA, USA) and placental alkaline phosphatase (PLAP) monoclonal antibody (5B1, Thermo Fisher Scientific). The PVDF membranes were thereafter incubated for 1 h at room temperature. Probed PVDF membranes were thereafter washed in Tris buffer saline (pH 7.6) and incubated with the exosome-validated secondary conjugated horseradish peroxidase antibody (System Biosciences, CA, USA) at room temperature for 1 h. Finally, the PVDF membranes were incubated with the chemiluminescent substrate and subsequently visualised using the ChemiDoc^®^ Gel Documentation System (Bio-Rad Laboratories, Hercules, CA, USA).

#### 3.5.3. Transmission Electron Microscopy

Isolated exosomes were immobilised on a continuous carbon grid. The grids were thereafter negatively stained with 2% uranyl acetate. The morphology of the exosomes was examined using a JEOL 1010 transmission electron microscope (JEOL, Peabody, MA, USA).

### 3.6. Quantification of Total and Placenta-Derived Exosomes

Total and placenta-derived exosomes were determined as per our previous publication [14]. Total exosomes in maternal circulation were quantified by the ELISA detection of immunoreactive exosomal CD63 (ExoELISA™, System Biosciences, Mountain View, CA, USA), as per the manufacturer’s instructions. Briefly, 35 µg of exosomal protein was immobilised onto the microtiter plate for 12 h at 37 °C using exosome binding buffer. Following incubation, microtiter plates were washed with buffer and incubated with the exosome-specific primary antibody (CD63) at room temperature for 1 h. Thereafter, the microtiter plate was washed and incubated with the secondary antibody (1:5000) at room temperature for 1 h. Finally, the microtiter plates were washed and incubated with Tetramethylbenzidine ELISA substrate with agitation for 45 min. Termination of the reaction was achieved by the addition of the stop buffer followed by measuring the absorbance at 450 nm. The number of exosomes/mL was extrapolated from a standard curve that was generated using the calibrated exosome standard which was supplied. 

Placenta-derived exosomes were quantified by the detection of human placental alkaline phosphatase utilising a commercial ELISA kit (Elabscience, E-EL-H1976, WuHan, PRC), as per the manufacturer’s instructions. Briefly, 35 µg of exosomal protein was added to the pre-coated plates containing the primary PLAP-specific antibody, followed by incubation at 37 °C for 90 min. Post-incubation plates were washed with buffer followed by the addition of 50 μL of HRP-conjugate and incubation at 37 °C for 30 min. Finally, the microtiter plates were washed and incubated with 50 μL of substrate A and B at 37 °C for 20 min. A total of 50 μL of stop solution was added to stop the reaction at room temperature for 2 min and the absorbance was subsequently measured at 450 nm. The exosomal PLAP-quantified inb each sample was expressed as pg/mL plasma.

### 3.7. NanoString nCounter System miRNA Assay

Exosomal RNA (115 ng) from each sample group was analysed utilising the NanoString nCounter SPRINT Profiler (NanoString Technologies^®^, Inc., Washington, DC, USA) as previously described [14]. In brief, a species-specific tag sequence (nCounter miRtag) was ligated onto exomiRs via thermally controlled splinted ligation. Enzymatic purification was used to remove unligated miRtags. The miR tagged mature miRNAs were thereafter hybridised using the nCounter Human (V3) miRNA Expression Assay Code Set at 65 °C for 16 h followed by sample loading into the nCounter SPRINT^TM^ cartridge and analysis using the nCounter^®^ SPRINT Profiler (NanoString Technologies^®^, Inc., Washington, DC, USA). Magnetic bead separation, liquid transfers and immobilisation of molecular labels on the sample surface were done by the instrument prior to the detection of specific miRNA probes. Quantification of the reporter probes was done for each sample by the nCounter Digital Analyzer which was subsequently analysed using the nSolver Software (V 4.0). Before the data were normalised, the nCounter data imaging QC metrics were validated using the nSolver program by statistically analysing the imaging, binding density, positive control linearity and positive control limit of detection. Additionally, there were no significant discrepancies between the fields of view attempted and the fields of view counted. The binding density for the samples was within the recommended range, i.e., between 0.20 and 0.74. The normalisation factor used by the nSolver program automatically validates data for technical noise such as variations in hybridisation, purification and binding efficiency. Code count normalisation was conducted using the geometric mean of the positive controls and the mean of the negative controls was used to determine the background. Exosomal RNA was normalised to the geometric mean of five housekeeping miRNA controls (RPLP0, RPL19, B2M, GAPDH and ACTB) and spike-in miRNA (osa-miR422, osa-miR414, osa-miR254, osa-miR248 and osa-miR159a) which were included in the assay. All data were finally normalised to the total miRNA count.

### 3.8. Statistical Data Analysis

All data analyses and graphical representations were performed and generated in GraphPad Prism 6.0 (CA, USA) and nSolver Analysis Software 4.0.7 (NanoString Technologies Inc, Washington, DC, USA). Exosomal miRNA ratio analysis between groups was done based on an arbitrary |fold change| ≥ 1.5 with all expression data represented in Log2 scale using the nSolver analysis software. All functional Kyoto Encyclopedia of Genes and Genomes (KEGG) and Gene Ontology (GO) analyses were performed using DIANA miRPATH (V3.0) together with DIANA-microT-CDS 5.0 algorithms (interactions with a *p*-value <0.001 were considered).

## 4. Conclusions

The known similarities between the PME and TME suggest that constituents of these microenvironments are involved in comparable molecular processes which control trophoblast and tumour cellular functions, respectively. In the present study, we show that exosomes, a key constituent of the PME, are involved in targeting immune, trophoblast invasion, migration and angiogenic biological processes in pregnancy, all of which coincide with tumour biological processes. Moreover, the computational merging and meta-analytical pathway analysis of the identified maternal exomiRs and their differential expression during pregnancy articulate the possible parallel biological links between placental and tumour development which will ultimately converge towards understanding the dynamic interactions involved in cancer metastasis and immunity. Importantly, we highlight key pregnancy-associated exomiRs which can be strategically repurposed towards developing effective cancer treatment. Therefore, our future studies will be focused on validating and repurposing exosomal mechanisms in pregnancy towards a cure for cancer as well as other immune-associated diseases. 

## Figures and Tables

**Table 1:** Clinical Characteristics of Participants.

Variables	Normotensive Pregnant Women	
	≤33 Weeks (*n* = 5)	≥34 Weeks (*n* = 5)
Age (years)	30.43 ± 1.1	33.12 ± 1.1
Weight (kg)	65.88 ± 3.25	67.57 ± 2.22
Height (cm)	155.77 ± 4.12	157.66 ± 4.55
BMI	25.98 ± 2.14	26.95 ± 2.99
Gestational Age (weeks)	30.11 ± 1.56 (26–33)	38 ± 1.9 (34–38)
Systolic/diastolic blood pressure (mm Hg)	123/81 ± 2.22/1.88(90–120/50–80)	119/79 ± 2.1/4.3 (90–120/50–80)
Urine Protein (mg/dL)	ND	ND

ND = not detected. All values are represented by mean ± SEM. All pregnancies were singleton without intrauterine infection or any other medical condition. The patients recruited had a parity of 1 ± 1.

**Table 2:** Computational Merging and Meta-Analytical Pathway Analysis (DIANA mirPATH) of Differentially Expressed Exosomal miRNA in Normotensive Pregnancies.

Exosomal miRNA Identified	Mean Expression Values (Log2)		Kyoto Encyclopedia of Genes and Genomes Analysis (*p* ≤ 0.05)			
	Normotensives (30 ± 1 Weeks)	Normotensives (38 ± 1 Weeks)	Computational DIANA miRPath microT-CDS Target Pathway	*p* Value	Target Genes	Target Physiological Function
hsa-miR-451a	11.06 ± 0.03	9.95 ± 0.01	Estrogen signaling (hsa04915)	3.0 × 10^−3^	ATF2	Cell cycle regulation, Proapoptotic protein regulation
hsa-miR-302d-3p	7.54 ± 0.21	9.24 ± 0.81	Estrogen signaling (hsa04915)	8.9 × 10^−5^	ESR1, ATF6B, CREB5, CREB1, SOS1, KCNJ6, SHC4, PIK3CA	Cell cycle regulation, Proapoptotic protein regulation
			Proteoglycans in cancer (hsa05205)	1.0 × 10^−3^	ESR1, PDCD4, SMAD2, SDC1, PTCH1, ROCK2, RDX, ERBB3, FZD3, ANK2, TFAP4, SOS1, PIK3CA, CD44	Cell migration and Invasion, Cell growth and survival
hsa-miR-223-3p	4.95 ± 0.1	5.5 ± 0.03	Mucin-type O-Glycan biosynthesis (hsa00512)	2.5 × 10^−6^	GALNT7, GALNT18	Protein secretion, stability, processing and function

ND = not detected. All values are represented by mean ± SEM. All functional Kyoto Encyclopedia of Genes and Genomes analyses were performed using DIANA miRPATH (V3.0) together with DIANA-microT-CDS 5.0 algorithms (interactions with a *p*-value ≤ 0.05 were considered).

## Supplementary Materials

Supplementary materials can be found at https://www.mdpi.com/1422-0067/21/15/5384/s1. Supplementary Table S1 lists the Log2 Exosomal miRNA Expression values of exomiRs with an arbitrary |fold change| ≥ 1.5.
