# Supplementary material for: Exosomal MicroRNAs in Pregnancy Provides Insight into a Possible Cure for Cancer
Source: Int J Mol Sci. 2020 Jul 29;21(15):5384. doi: 10.3390/ijms21155384 (PMC7432616; doi:10.3390/ijms21155384)
Supplement: Supplementary file 1 [file ijms-21-05384-s001.pdf]

Supplementary Table 1: Log2 Exosomal miRNA Expression values of exomiRs in pregnant woman (33 ± 1 weeks and 38 ± 1 weeks, n=5) with an arbitrary |fold change| ≥ 1.5.

miRNA	Mean Expression Values (Log2) (33 ±1 Weeks)	Mean Expression Values (Log2) (38 ±1 Weeks)
hsa-miR-451a	11.06 ±0.07	9.95 ±0.09
hsa-miR-302d-3p	7.54 ±0.06	9.24 ±0.01
hsa-miR-223-3p	4.95 ±0.11	5.5 ±0.05
hsa-miR-520h	2 ±0.00	4.4 ±0.00
hsa-miR-122-5p	2 ±0.09	4.3 ±0.11
hsa-miR-4755-5p	2 ±0.11	4 ±0.09
hsa-miR-219b-3p	2 ±0.10	4 ±0.08
hsa-miR-612	1.8 ±0.08	3.4 ±0.01
hsa-miR-2116-5p	1.7 ±0.01	3.2 ±0.01
hsa-miR-548g-3p	1.8 ±0.03	3.3 ±0.01
hsa-miR-92a-3p	1.9 ±0.00	3.5 ±0.04
hsa-miR-765	1.8 ±0.01	3.2 ±0.01
hsa-miR-573	1.6 ±0.08	2.78 ±0.04
hsa-miR-371a-5p	1.3 ±0.08	2.2 ±0.01
hsa-miR-639	1.8 ±0.01	3.04 ±0.09
hsa-miR-548e-5p	1.7 ±0.00	2.85 ±0.11
hsa-miR-196a-5p	1.71 ±0.09	2.86 ±0.07
hsa-miR-513b-5p	1.69 ±0.09	2.8 ±0.00
hsa-miR-130a-3p	1.65 ±0.07	2.75 ±0.11
hsa-miR-148b-3p	1.66 ±0.10	2.7 ±0.01
hsa-miR-3202	1.65 ±0.00	2.6 ±0.04
hsa-miR-764	1.66 ±0.11	2.61 ±0.04
hsa-miR-4707-3p	1.64 ±0.05	2.59 ±0.02
hsa-miR-499a-5p	1.5 ±0.05	2.35±0.04
hsa-miR-575	1.6 ±0.05	2.47 ±0.11
hsa-miR-1185-1-3p	1.5 ±0.08	2.3 ±0.08
hsa-miR-548i	1.8 ±0.01	2.75 ±0.11
hsa-miR-574-5p	1.9 ±0.00	2.88 ±0.09
hsa-miR-649	1.5 ±0.00	2.25 ±0.09
hsa-miR-585-3p	2.5 ±0.01	1 ±0.00
hsa-miR-1286	5.1 ±0.04	2 ±0.00
hsa-miR-1296-5p	2.54 ±0.05	1 ±0.01
hsa-miR-148a-3p	2.55 ±0.03	1 ±0.01
hsa-miR-150-5p	2.54 ±0.04	1 ±0.04
hsa-miR-181a-3p	2.54 ±0.04	1 ±0.03
hsa-miR-3168	2.54 ±0.00	1 ±0.01
hsa-miR-369-3p	2.54 ±0.10	1 ±0.04
hsa-miR-660-3p	2.54 ±0.11	1 ±0.08
hsa-miR-802	2.54 ±0.10	1 ±0.11
hsa-miR-376a-2-5p	2.68 ±0.11	1.05 ±0.13
hsa-miR-551a	2.66 ±0.12	1.04 ±0.09
hsa-miR-1976	2.68 ±0.08	1.04 ±0.11
hsa-miR-548a-3p	2.67 ±0.11	1.04 ±0.13
hsa-miR-1287-5p	2.5 ±0.01	0.98 ±0.10
hsa-miR-320b	2.5 ±0.01	0.98 ±0.11
hsa-miR-33b-5p	2.5 ±0.11	0.98 ±0.01
hsa-miR-873-5p	2.5 ±0.11	0.98 ±0.01
hsa-miR-510-3p	5.31 ±0.10	2 ±0.00
hsa-miR-20a-5p+hsa-miR-20b-5p	5.4 ±0.06	2.02 ±0.02
hsa-miR-345-3p	2.5 ±0.05	0.92 ±0.03
hsa-miR-5010-3p	2.5 ±0.01	0.92 ±0.12
hsa-miR-548l	2.5 ±0.02	0.92 ±0.02
hsa-miR-1827	2.4 ±0.05	0.88 ±0.11
hsa-miR-1185-5p	2.5 ±0.01	0.9 ±0.12

hsa-miR-1245b-3p	2.5 ±0.11	0.9 ±0.06
hsa-miR-382-3p	2.5 ±0.07	0.9 ±0.09
hsa-miR-563	2.3 ±0.12	0.9 ±0.01
hsa-miR-589-5p	4.24 ±0.15	1.5 ±0.01
hsa-miR-629-5p	4.24 ±0.09	1.5 ±0.08
hsa-miR-26b-5p	4 ±0.01	1.4 ±0.09
hsa-miR-3150b-3p	3.73 ±0.10	1.3 ±0.12
hsa-miR-323a-3p	3.73 ±0.11	1.3 ±0.09
hsa-miR-548j-3p	3.73 ±0.01	1.3 ±0.10
hsa-miR-21-5p	4 ±0.06	1.36 ±0.10
hsa-miR-412-3p	4 ±0.07	1.34 ±0.11
